# Complete genome sequence of *Spirosoma linguale* type strain (1^T^)

**DOI:** 10.4056/sigs.741334

**Published:** 2010-03-30

**Authors:** Kathleen Lail, Johannes Sikorski, Elizabeth Saunders, Alla Lapidus, Tijana Glavina Del Rio, Alex Copeland, Hope Tice, Jan-Fang Cheng, Susan Lucas, Matt Nolan, David Bruce, Lynne Goodwin, Sam Pitluck, Natalia Ivanova, Konstantinos Mavromatis, Galina Ovchinnikova, Amrita Pati, Amy Chen, Krishna Palaniappan, Miriam Land, Loren Hauser, Yun-Juan Chang, Cynthia D. Jeffries, Patrick Chain, Thomas Brettin, John C. Detter, Andrea Schütze, Manfred Rohde, Brian J. Tindall, Markus Göker, Jim Bristow, Jonathan A. Eisen, Victor Markowitz, Philip Hugenholtz, Nikos C. Kyrpides, Hans-Peter Klenk, Feng Chen

**Affiliations:** 1 DOE Joint Genome Institute, Walnut Creek, California, USA; 2 DSMZ – German Collection of Microorganisms and Cell Cultures GmbH, Braunschweig, Germany; 3 Los Alamos National Laboratory, Bioscience Division, Los Alamos, New Mexico, USA; 4 Biological Data Management and Technology Center, Lawrence Berkeley National Laboratory, Berkeley, California, USA; 5 Oak Ridge National Laboratory, Oak Ridge, Tennessee, USA; 6 Lawrence Livermore National Laboratory, Livermore, California, USA; 7 HZI – Helmholtz Centre for Infection Research, Braunschweig, Germany; 8 University of California Davis Genome Center, Davis, California, USA

**Keywords:** psychroactive, oligotrophic, aerobic, ringlike morphology, non-pathogenic, free-living, *Cytophagaceae*, GEBA

## Abstract

*Spirosoma linguale* Migula 1894 is the type species of the genus. *S. linguale* is a free-living and non-pathogenic organism, known for its peculiar ringlike and horseshoe-shaped cell morphology. Here we describe the features of this organism, together with the complete genome sequence and annotation. This is only the third completed genome sequence of a member of the family *Cytophagaceae*. The 8,491,258 bp long genome with its eight plasmids, 7,069 protein-coding and 60 RNA genes is part of the *** G****enomic* *** E****ncyclopedia of* *** B****acteria and* *** A****rchaea * project.

## Introduction

Strain 1^T^ (= DSM 74 = ATCC 33905 = LMG 10896) is the type strain of the species *Spirosoma linguale*, which is the type species of the genus *Spirosoma*. The genus currently consists of five species [1]. Strain 1^T^ is reported to be isolated from a laboratory water bath (websites of DSMZ and ATCC), however, a proper reference could not be identified. Another strain of *S. linguale* was isolated from fresh water from deep wells in Long Beach, California, USA [2]. Other strains from the genus *Spirosoma* were isolated from high arctic permafrost soil in Norway [3], soil from a ginseng field in Pocheon province, South Korea [4], and fresh water from the Woopo wetlands, South Korea [5]. This would allow the hypothesis that *S. linguale* is a free-living species with a worldwide distribution. The genus name *Spirosoma* derives from ‘spira’ from Latin meaning coil combined with ‘soma’, Latin for ‘body’, resulting in ‘coiled body’ [1]. *Spirosoma* was the first genus in the family *Spirillaceae* in Migula’s “System der Bakterien” [6]. The species name is effectively published by Migula in 1894 [7] and validly published by Skerman in 1980 [8]. Various taxonomic treatments have placed this organism either in the family “*Flexibacteraceae*” or the family *Cytophagaceae*. This would appear to be due to a number of nomenclatural problems. The family “*Flexibacteriaceae*” as outlined in TOBA 7.7 would include *Cytophaga hutchinsonii*, which is the type species of the genus *Cytophaga*, which, in turn is the type of the family *Cytophagaceae*, a name that may not be replaced by the family name “*Flexibacteriaceae”* as long as *Cytophaga hutchinsonii* is one of the included species. However, the topology of the 16S rDNA based dendrogram indicates that it may be possible to define a second family, including the genus *Spirosoma*, but excluding *Cytophaga hutchinsonii*. At the same time, the family *Cytophagaceae* may be defined to exclude the type species of the genus *Flexibacter* and members of the genus *Spirosoma*. It should also be remembered that the genus *Spirosoma* is the type of the family *Spirosomaceae* Larkin and Borrall 1978. At present the higher taxonomic ranks of this group of organisms lacks formal modern descriptions and circumscriptions making it difficult to make definitive statements that would hold over the next few years. Here we present a summary classification and a set of features for *S. linguale* 1^T^, together with the description of the complete genomic sequencing and annotation.

## Classification and features

Uncultured clone sequences in Genbank showed 96% or less sequence identity to the 16S gene sequence (AM000023) of strain *S. linguale* 1^T^. No reasonable sequence similarity (>87%) to any metagenomic survey were reported from the NCBI BLAST server (October 2009).

**Figure 1:**
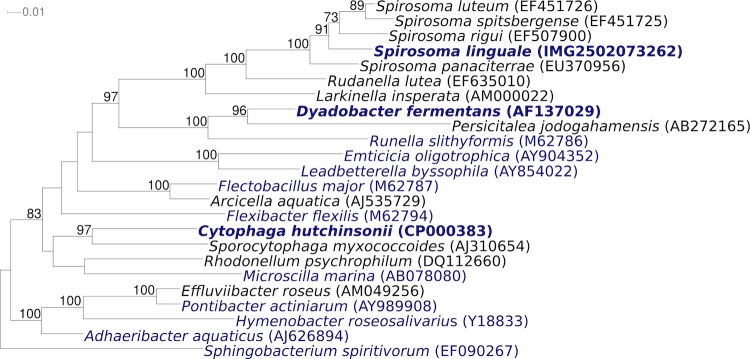
Phylogenetic tree highlighting the position of *S. linguale* 1^T^ and the type strains of the other species within the genus relative to the other type strains within the family *Cytophagaceae*. The tree was inferred from 1,320 aligned characters [9,10] of the 16S rRNA gene sequence under the maximum likelihood criterion [11] and rooted with the type strain of the family *Sphingobacteriaceae*. The branches are scaled in terms of the expected number of substitutions per site. Numbers above branches are support values from 1,000 bootstrap replicates if larger than 60%. Lineages with type strain genome sequencing projects registered in GOLD [12] are shown in blue, published genomes such as the one of *Dyadobacter fermentans* [13] in bold.

Figure 1 shows the phylogenetic neighborhood of for *S. linguale* 1^T^ in a 16S rRNA based tree. The sequences of the four identical 16S rRNA gene copies in the genome of *S. linguale* 1^T^ are also identical with the previously published 16S rRNA sequence generated from LMG 10896 (AM000023).

**Figure 2:**
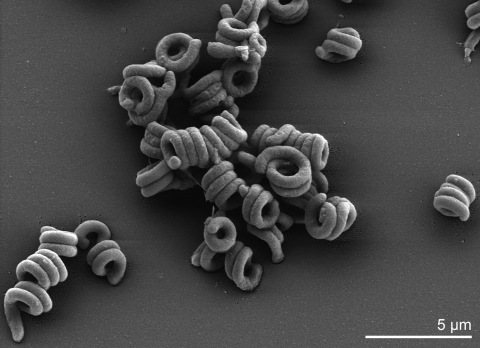
Scanning electron micrograph of *S. linguale* 1^T^

On TGEY medium [14], strain *S. linguale* 1^T^ forms mucoid, opaque, and smooth colonies with a yellowish nondiffusible pigment [15]. The colony size is 2-4 mm, circular, with entire margins and convex elevation. In broth, growth is aerobic (Table 1) with even turbidity and flaky sediment [15]. The Gram-negative cells have round ends and show vibroid, horseshoe, and ring-like shapes, as well as coils and spiral forms [Figure 2 and ref. 15]. The cell width is 0.5 – 1.0 µm, and the outer ring diameter is 1.5–3.0 µm. The cell length is 2.0–5.0 µm [22]. Reports on filaments are conflicting [15,22].

**Table 1:** Classification and general features of *S. linguale* 1^T^ according to the MIGS recommendations [16]

**MIGS ID**	**Property**	**Term**	**Evidence code**
	Current classification	Domain *Bacteria*	TAS [17]
		Phylum *Bacteroidetes*	TAS [18,19]
		Class *Sphingobacteria*	TAS [18,20]
		Order *Sphingobacteriales*	TAS [18,20]
		Family *Cytophagaceae*	TAS [8,21]
		Genus *Spirosoma*	TAS [3,7,8]
		Species *Spirosoma linguale*	TAS [7]
		Type strain 1	TAS [7,8]
	Gram stain	negative	TAS [15]
	Cell shape	vibroid, horseshoe, and ring-like shapes; spiral form	TAS [15]
	Motility	non-motile	TAS [15]
	Sporulation	nonsporulating	NAS
	Temperature range	5°C–39°C	TAS [5]
	Optimum temperature	20°C–30°C	TAS [5]
	Salinity	0-1.25% (w/v)	TAS [5]
MIGS-22	Oxygen requirement	aerobic	TAS [15]
	Carbon source	glycerol phosphate, succinate, tartrate, malonate	TAS [22]
	Energy source	carbohydrates	TAS [22]
MIGS-6	Habitat	Laboratory water bath	NAS
MIGS-15	Biotic relationship	free-living	NAS
MIGS-14	Pathogenicity	not reported	NAS
	Biosafety level	1	TAS [23]
	Isolation	not reported	NAS
MIGS-4	Geographic location	Germany	NAS
MIGS-5	Sample collection time	not reported	
MIGS-4.1 MIGS-4.2	Latitude Longitude	not reported	
MIGS-4.3	Depth	not reported	
MIGS-4.4	Altitude	not reported	

Evidence codes - IDA: Inferred from Direct Assay (first time in publication); TAS: Traceable Author Statement (i.e., a direct report exists in the literature); NAS: Non-traceable Author Statement (i.e., not directly observed for the living, isolated sample, but based on a generally accepted property for the species, or anecdotal evidence). These evidence codes are from of the Gene Ontology project [24]. If the evidence code is IDA, then the property was directly observed for a live isolate by one of the authors, or an expert mentioned in the acknowledgements.

Strain *S. linguale* 1^T^ produces oxidatively acid from arabinose, ribose, xylose, rhamnose, fructose, galactose, glucose, mannose, α-methyl-D-glucoside, salicin, cellobiose, lactose, maltose, melibiose, sucrose, trehalose, raffinose, dextrin and inulin, but not from sorbose, glycerol, erythritol, dulcitol, mannitol, and sorbitol [22]. On the enzymatic level, strain *S. linguale* 1^T^ is positive for oxidase, catalase, ONPG-reaction, and, albeit weakly, for phosphatase, but negative for urease, lecithinase, lysine decarboxylase, phenylalanine deaminase, and hemolysin, indole, methyl red, Voges-Proskauer, NO_3_ reduction and H_2_S reactions [22]. Strain *S. linguale* 1^T^ hydrolyzes esculin, tributyrin, gelatin, and, less well, starch and casein, but not cellulose and chitin [22]. It utilizes for growth on basal medium [25] glycerol phosphate, succinate, tartrate, and malonate as single carbon source, but not acetate, benzoate, citrate, formate, methylamine, propionate, and methanol [22]. Strain *S. linguale* 1^T^ grows well on nutrient agar, nutrient agar + 5% sucrose, *Microcyclus-Spirosoma* agar, and yeast extract tryptone agar, weakly on peptonized milk agar, blood, and chocolate, and not on eosin methylene blue agar, phenol red mannitol salt agar, phenyl ethyl alcohol agar, trypticase soy agar (TSA), TSA + 3% glucose, TSA + 3% sucrose, McConkey, bismuth sulfide agar, and *Salmonella-Shigella* agar [22]. Strain *S. linguale* 1^T^ is susceptible to actinomycin D (100 µg/ml), ampicillin (10 µg), aureomycin (15 µg), carbenicillin (50 µg), erythromycin (15 µg), furadantin/macrodantin (300 µg), gentamicin (10 µg), kanamycin (30 µg), mitomycin C (1 µg/ml), neomycin (30 µg), penicillin G (10 units), streptomycin (10 µg), sulfamethoxyzole/trimethopterin (25 µg), sulfathiazole (300 µg), and tetracycline(30 µg), but resistant to colistin (10 µg), polymixin B (300 units), and triple sulfa (1 mg) [22].

### Chemotaxonomy

Earlier studies report C_16:1_ to be the dominant fatty acid (47.9%), followed by iso-C_17:0_ (20.1%), C_16:0_ (14.2%), iso-C_15:0_ (11.0%) and iso-C_13:0_ (3.4%). Anteiso and hydroxy fatty acids are each below 2.1% [26]. The fatty acids comprise a complex mixture of straight chain saturated and unsaturated fatty acids, together with iso-branched and 3-hydroxylated iso-branched fatty acids. The fatty acids comprise iso-C_13:0_ (2.2%), iso-C_15:0_ (9.3%), iso-C_15:0_ 3-OH (3.4%), anteiso-C_15:0_ (2.6%), C_16:0_ (3.6%), C_16:0_ 3-OH (2.2%), C_16:1_ ω 5c (22.2%), C_17:0_ 2-OH (1.0%), iso-C_17:0_ 3-OH (8.6%), iso-C_13:0_ (2.2%), C_17:1_ ω 9c (1.2%) and C_16:1_ ω 7c and/or iso-C_15:0_ 2-OH (42.4%). The polar lipids comprise phosphatidylethanolamine and a number of lipids and amino lipids that were not further characterized. The fatty acid pattern is typical of the evolutionary group currently defined as the phylum *Bacteroidetes*. Furthermore the presence of phosphatidylethanolamine as the predominant/sole diglyceride based phospholipid is also typical of the vast majority of the phylum *Bacteroidetes*. Limited detailed studies indicate that this phospholipid contains both saturated and unsaturated straight chain fatty acids. Hydroxylated fatty acids are not present in this compound. In contrast, the limited studies on the amino lipids of *Flavobacterium johnsoniae* indicate that they are amino acid based, with a 3-OH fatty acid in amide linkage with a free amino group of the amino acid. The 3-OH fatty acid is further esterified with either a non-hydroxylated fatty acid, or with a second hydroxylated fatty acid. The presence of lipids that did not stain further running in proximity with the major aminolipid may also be indicative of capnines. The failure to resolve the fatty acids reported by the MIDI Sherlock MIS system as “summed feature 4” C_16:1ω7c_/iso C_15:0 _2-OH is problematic for genomics, since it either indicates that two mechanisms of introducing double bonds into fatty acids are present (C_16:1ω5c _and C_16:1ω7c_) or a fatty acid 2-hydroxylase is present. Furthermore, the distribution of 3-OH and 2-OH fatty acids among the amino- and non-staining lipids may also be characteristic. The main isoprenoid quinone is MK-7 (91.5%), followed by MK-8 (7.2%) and MK-6 (1.3%) [26].

## Genome sequencing and annotation

### Genome project history

This organism was selected for sequencing on the basis of its phylogenetic position, and is part of the *** G****enomic* *** E****ncyclopedia of* *** B****acteria and* *** A****rchaea * project. The genome project is deposited in the Genome OnLine Database [12] and the complete genome sequence is deposited in GenBank. Sequencing, finishing and annotation were performed by the DOE Joint Genome Institute (JGI). A summary of the project information is shown in Table 2.

**Table 2:** Genome sequencing project information

**MIGS ID**	**Property**	**Term**
MIGS-31	Finishing quality	Finished
MIGS-28	Libraries used	Two Sanger libraries: 8kb pMCL200 and fosmid pcc1Fos One 454 pyrosequence and one Solexa standard library
MIGS-29	Sequencing platforms	ABI3730, 454 GS FLX, Illumina GA
MIGS-31.2	Sequencing coverage	10.1× Sanger; 18.4× pyrosequence
MIGS-30	Assemblers	Newbler 1.1.02.15, phrap
MIGS-32	Gene calling method	Prodigal, GenePRIMP
	INSDC ID	CP001769 (chromosome) CP001770-77 (plasmids)
	Genbank Date of Release	January 13, 2010
	GOLD ID	Gc01186
	NCBI project ID	28817
	Database: IMG-GEBA	2501939635
MIGS-13	Source material identifier	DSM 74
	Project relevance	Tree of Life, GEBA

### Growth conditions and DNA isolation

*S. linguale* 1^T^, DSM 74, was grown in DSMZ medium 7 [27] at 28°C. DNA was isolated from 0.5-1 g of cell paste using Qiagen Genomic 500 DNA Kit (Qiagen, Hilden, Germany) with cell lysis modification st/L [28] and one hour incubation at 37°C.

### Genome sequencing and assembly

The genome was sequenced using a combination of Sanger and 454 sequencing platforms. All general aspects of library construction and sequencing can be found at http://www.jgi.doe.gov/. 454 Pyrosequencing reads were assembled using the Newbler assembler version 1.1.02.15 (Roche). Large Newbler contigs were broken into 9,401 overlapping fragments of 1,000 bp and entered into assembly as pseudo-reads. The sequences were assigned quality scores based on Newbler consensus q-scores with modifications to account for overlap redundancy and to adjust inflated q-scores. A hybrid 454/Sanger assembly was made using the parallel phrap assembler (High Performance Software, LLC). Possible mis-assemblies were corrected with Dupfinisher [29] or transposon bombing of bridging clones (Epicentre Biotechnologies, Madison, WI). Gaps between contigs were closed by editing in Consed, custom primer walk or PCR amplification. A total of 974 Sanger finishing reads were produced to close gaps, to resolve repetitive regions, and to raise the quality of the finished sequence. Illumina reads were used to improve the final consensus quality using an in-house developed tool (the Polisher). The error rate of the completed genome sequence is less than 1 in 100,000. Together all sequence types provided 28.5× coverage of the genome. The final assembly contains 87,186 Sanger and 666,973 pyrosequence reads.

### Genome annotation

Genes were identified using Prodigal [30] as part of the Oak Ridge National Laboratory genome annotation pipeline, followed by a round of manual curation using the JGI GenePRIMP pipeline [31]. The predicted CDSs were translated and used to search the National Center for Biotechnology Information (NCBI) nonredundant database, UniProt, TIGRFam, Pfam, PRIAM, KEGG, COG, and InterPro databases. Additional gene prediction analysis and manual functional annotation was performed within the Integrated Microbial Genomes Expert Review (IMG-ER) platform [32].

## Genome properties

**Figure 3:**
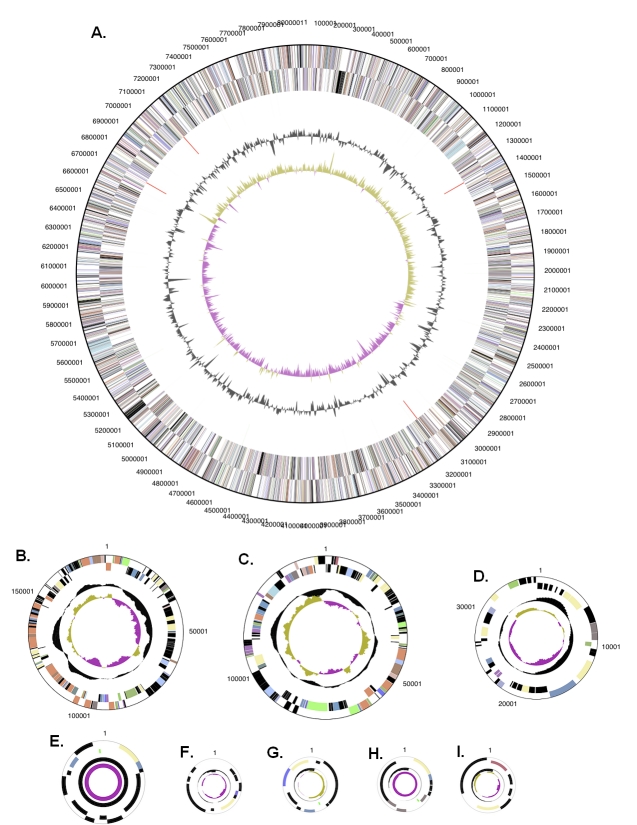
Graphical circular map of the chromosome (A) and the eight plasmids: pSLIN01 (B), pSLIN02 (C), pSLIN03 (D), pSLIN04 (E), pSLIN05 (F), pSLIN06 (G), pSLIN07 (H), pSLIN08 (I). Plasmids not drawn to scale. From outside to the center: Genes on forward strand (color by COG categories), Genes on reverse strand (color by COG categories), RNA genes (tRNAs green, rRNAs red, other RNAs black), GC content, GC skew.

The genome consists of a 8,078,757 bp long chromosome and eight plasmids with 6,072 to 189,452 bp length (Table 3 and Figure 3). Of the 7,129 genes predicted, 7,069 were protein-coding genes, and 60 RNAs; 131 pseudogenes were also identified. The majority of the protein-coding genes (61.5%) were assigned with a putative function while those remaining were annotated as hypothetical proteins. The distribution of genes into COGs functional categories is presented in Table 4.

**Table 3:** Genome Statistics

**Attribute**	**Value**	**% of Total**
Genome size (bp)	8,491,258	100.00%
DNA coding region (bp)	7,518,086	88.54%
DNA G+C content (bp)	4,258,276	50.15%
Number of replicons	9	
Extrachromosomal elements	8	
Total genes	7,129	100.00%
RNA genes	60	0.84%
rRNA operons	4	
Protein-coding genes	7,069	99.16%
Pseudo genes	131	1.84%
Genes with function prediction	4,386	61.52%
Genes in paralog clusters	1,713	2.71%
Genes assigned to COGs	4,306	60.40%
Genes assigned Pfam domains	4,519	63.39%
Genes with signal peptides	2,271	41.86%
Genes with transmembrane helices	1,606	22.53%
CRISPR repeats	2	

**Table 4:** Number of genes associated with the general COG functional categories

**Code**	**Value**	**%age**	**Description**
J	173	2.4	Translation, ribosomal structure and biogenesis
A	1	0.0	RNA processing and modification
K	398	6.6	Transcription
L	251	3.6	Replication, recombination and repair
B	1	0.0	Chromatin structure and dynamics
D	32	0.5	Cell cycle control, mitosis and meiosis
Y	0	0.0	Nuclear structure
V	161	2.3	Defense mechanisms
T	380	5.4	Signal transduction mechanisms
M	411	5.8	Cell wall/membrane biogenesis
N	14	0.2	Cell motility
Z	0	0.0	Cytoskeleton
W	0	0.0	Extracellular structures
U	68	1.0	Intracellular trafficking and secretion
O	161	2.3	Posttranslational modification, protein turnover, chaperones
C	223	3.2	Energy production and conversion
G	383	5.4	Carbohydrate transport and metabolism
E	297	4.2	Amino acid transport and metabolism
F	80	1.1	Nucleotide transport and metabolism
H	174	2.5	Coenzyme transport and metabolism
I	162	2.3	Lipid transport and metabolism
P	277	3.9	Inorganic ion transport and metabolism
Q	121	1.7	Secondary metabolites biosynthesis, transport and catabolism
R	611	8.6	General function prediction only
S	424	6.0	Function unknown
-	2,823	39.9	Not in COGs
